# Ketamine/xylazine and barbiturates modulate microglial morphology and motility differently in a mouse model

**DOI:** 10.1371/journal.pone.0236594

**Published:** 2020-08-06

**Authors:** Ines Hristovska, Franck Verdonk, Jean-Christophe Comte, Eileen S. Tsai, Virginie Desestret, Jérôme Honnorat, Fabrice Chrétien, Olivier Pascual

**Affiliations:** 1 Equipe Synaptopathies et Autoanticorps (SynatAc), Institut NeuroMyoGène, INSERM U1217/UMR CNRS 5310, Lyon, France; 2 Université Claude Bernard Lyon 1, Université de Lyon, Lyon, France; 3 Unité Neuropathologie Expérimentale, Département Infection et Epidémiologie, Institut Pasteur, Paris, France; 4 Department d’anesthésiologie et de Soins Intensifs, Hôpital Saint Antoine, Assistance Publique-Hôpitaux de Paris, Paris, France; 5 Sorbonne Université, Paris, France; 6 Department of Anesthesiology, Perioperative and Pain Medicine, Stanford University School of Medicine, Stanford, California, United States of America; 7 Equipe Processus d’oubli et Dynamique Corticale, Centre de Recherche en Neuroscience de Lyon (CRNL), INSERM U1028, CNRS UMR5292, Lyon, France; 8 Centre maladies rares sur les syndromes neurologiques paranéoplasiques, Hospices Civils de Lyon, Lyon, France; 9 Université Paris Descartes, Sorbonne Paris Cité, Paris, France; 10 Laboratoire Hospitalo-Universitaire de Neuropathologie, Centre Hospitalier Sainte Anne, Paris, France; Albany Medical College, UNITED STATES

## Abstract

Microglia, the resident immune cells of the brain, are highly ramified and motile and their morphology is strongly linked to their function. Microglia constantly monitor the brain parenchyma and are crucial for maintaining brain homeostasis and fine-tuning neuronal networks. Besides affecting neurons, anesthetics may have wide-ranging effects mediated by non-neuronal cells and in particular microglia. We thus examined the effect of two commonly used anesthetic agents, ketamine/xylazine and barbiturates, on microglial motility and morphology. A combination of two-photon *in vivo* imaging and electroencephalography (EEG) recordings in unanesthetized and anesthetized mice as well as automated analysis of *ex vivo* sections were used to assess morphology and dynamics of microglia. We found that administration of ketamine/xylazine and pentobarbital anesthesia resulted in quite distinct EEG profiles. Both anesthetics reduced microglial motility, but only ketamine/xylazine administration led to reduction of microglial complexity *in vivo*. The change of cellular dynamics *in vivo* was associated with a region-dependent reduction of several features of microglial cells *ex vivo*, such as the complexity index and the ramification length, whereas thiopental altered the size of the cytoplasm. Our results show that anesthetics have considerable effects on neuronal activity and microglial morphodynamics and that barbiturates may be a preferred anesthetic agent for the study of microglial morphology. These findings will undoubtedly raise compelling questions about the functional relevance of anesthetics on microglial cells in neuronal physiology and anesthesia-induced neurotoxicity.

## Introduction

Anesthetics are widely administered in animal research studies. They are commonly used to generate a reversible brain state allowing surgery and *in vivo* imaging of animals with fewer motion artifacts and less stress during contention. Excess anesthetics can also be used for euthanasia allowing further anatomical studies, such as immunohistochemistry. As major pharmacological modulators of neuronal activity, anesthetic agents may alter animal neurophysiology. Anesthetic-specific effects are mediated through a combination of channels and determined circuits and result in distinct neuronal activity patterns depending on the anesthetic [1–3]. Furthermore, recent studies revealed detrimental neurotoxic effects of general anesthetics that lead to morpho-functional changes in the CNS and impaired neurocognitive performance [1]. The mechanisms leading to modulation of neuronal activity and neurotoxicity are not clearly understood and little consideration has been given to mechanisms mediated through the action of glial cells. Several recent studies have shown that general anesthetics may affect glial cell morphology and function [4,5], but more research is needed.

Microglial cells, are the resident immune cells of the brain and are crucial in maintaining brain homeostasis [6]. In physiological conditions, microglial cells are highly ramified and dynamic, continuously surveying the surrounding parenchyma in an activity-dependent manner [7–9]. In addition, recent studies attribute essential functions to microglia, including maintenance of synaptic properties, regulation of neuronal activity and network synchronization, and involvement in learning and memory [10,11]. The effect of anesthesia on microglial morphodynamics and function has just recently started to be investigated and reveals quite contradictory findings.

Isoflurane effects have been largely studied on microglial cells *in situ* and *in vivo*, but no consensus has been reached with regards to its effect on microglial morphodynamics [5,12,13]. Ketamine/xylazine and urethane were found to increase microglial process area and process surveillance territory [5], but this was not corroborated by other studies [12,13]. These discrepancies between studies may account for different preparations and/or microglial states. Furthermore, the fact that process ramification and motility can evolve in opposite ways following fentanyl cocktail administration brings additional confusion [14]. Anesthetics were also tested on microglial inflammatory response. Ketamine induced the activation of microglial cells in the retrosplenial cortex, but not in the cingulate cortex of rats [15], while pentobarbital administration for 24 hours in cell culture changed microglial morphology from a ramified to a rounded shape [16]. However, neuroleptic anesthetics targeting dopamine and opioid receptors did not cause any activation of microglial cells in the hippocampus of young adult mice [17].

Microglial morphology has been associated with its different roles in physiological and pathological conditions. For instance, reduction of microglial ramification, enlargement of cell bodies, and shortening and thickening of processes are characteristic of brain inflammation or injury [18,19]. These morphological changes are associated with pro-inflammatory cytokine secretion, phagocytic activity and neuronal synchrony decrease [11,20]. On the other hand, microglia may also hyper-ramify in response to sensory deprivation [21], stress [22] and accelerated aging [23], but this phenotype and its potential consequences have been much less described. Considering the importance of microglial cells in brain homeostasis and the routine usage of anesthetics, a detailed description of the effects of anesthetics on microglial morphology and motility is needed.

In our study, we combined immunohistochemistry and two-photon *in vivo* imaging to study the effects of two anesthetics on microglial motility and several parameters of microglial morphology. We chose two commonly used anesthetic agents: pentobarbital/thiopental-based anesthetics (GABA_A_R agonist) and ketamine-xylazine cocktail (NMDAR antagonist). These act on different neuronal targets and have different effects on neuronal activity. Our findings indicate that these anesthetics differentially affect microglial motility and morphology and that their action varies depending on the brain region considered.

## Materials and methods

### Animals

*In vivo* and *ex vivo* experiments used six to ten week-old male heterozygous CX3CR1^eGFP(+/-)^ mice that expressed enhanced green fluorescent protein (eGFP) under the control of CX3CR1 promoter. Mice were housed in individual cages with bedding and running wheels, normal light/dark conditions and food and water *ad libitum*. All experimental procedures were carried out in accordance with the French institutional guidelines and ethical committee and authorized by the local Ethics Committees: CEEA-55/CETEA-2015-0038 and the Ministry of National Education and Research (APAFIS#6449–116 2016052515127983 v1).

### *In vivo* experiments

#### Surgery and habituation

Mice were handled during one week prior to surgery. For surgery, mice were deeply anesthetized with isoflurane (3–4%, Isovet, Piramal Healthcare, UK Ltd.) and mounted in a stereotaxic apparatus (D. Kopf Instruments). To relieve post-operatory pain and inflammation, Carprofen (5mg/kg s.c.) was administered at the beginning of the surgery and the following two days. For transcranial imaging, a custom-made head plate implant was positioned on the left hemisphere and the skull was carefully thinned over the somatosensory cortex using a high-speed dental drill. For electrophysiological recordings, two EEG screws were inserted in the frontal and parietal cortex of the right hemisphere and two EMG electrodes were inserted in the neck muscles.

A custom-made restraint system was used during head-restraining habituation sessions. Our habituation protocol involved daily training sessions over 7–10 days lasting progressively longer (from 10 minutes to 4 hours). A reward of several drops of sweetened concentrated milk was administered at the beginning and end of each session. Mice were imaged at the end of the habituation sessions.

#### Treatment conditions

Two-photon imaging was performed in the somatosensory cortex in the same mice (n = 6) pre-anesthesia and during anesthesia. Thus, the same microglial cells were imaged in pre-anesthesia and subsequently during anesthesia. In the «anesthesia» condition, mice were injected intraperitoneally with either a mixture of ketamine (100mg/kg) and xylazine (10mg/kg) or pentobarbital (60mg/kg) dissolved in 0.9% saline.

#### Two-photon *in vivo* imaging microscopy

A two-photon microscope (Olympus) with a mode-locked Ti:Sapphire laser (Mai-Tai, Spectra-Physics) tuned to 900nm (excitation wavelength for eGFP) was used. eGFP-labeled microglia were imaged under a 20x water-immersion objective (0.95 N.A. Olympus). Fluorescence was detected using a 560nm dichroic mirror coupled to a 525/50nm emission filter and a photomultiplier tube in whole-field detection mode. Laser power during imaging was maintained below 20mW.

Microglial cells in the somatosensory cortex were imaged at least 15 minutes after general anesthesia. The imaging parameters corresponded to 200x200μm field of view and resolution of 521x521 pixels approximately. Microglia were imaged at a depth of 50–150 μm from the cortical surface and a typical recording lasted approximately 15–20 minutes (30–40 stacks). 26–37 consecutive Z-stack images were acquired every 30 seconds, 1μm/optical section.

#### EEG/EMG recordings

During the entire imaging session, the vigilance states were monitored using real-time EEG/EMG differential recordings amplifier (Model 3000, A-M systems). Signals were sampled at 1kHz. EEG was filtered in the frequency band [0.5Hz-300Hz], while EMG was filtered in the [10-500Hz] frequency band. EEG data were analyzed using a custom MATLAB^©^ software. Power spectra and probability distributions of EEG magnitude were estimated for the total duration of anesthesia. Time-frequency representation was performed with a 4s duration sliding FFT (fast Fourier transform) window and 0.5s step size.

#### Imaging analysis

Image processing and analysis were performed using custom-written MatLab^©^ software. From the original image, we manually delimited regions of interest containing the totality of only one microglial cell. In order to correct the drift in the x, y and z planes, each volume was registered to a reference volume (the first volume) using shift estimation from the cross-correlation peak by FFT. After realignment, standard deviation intensity projections of z stacks were created and used to generate 2D time-lapse movies.

For analyses of microglial complexity, we transformed the images into binary and calculated the Hausdorff fractal dimension, thus providing quantitative measure of the complexity of microglial cells. For each series of images, cell complexity was determined by averaging the complexity values obtained for each image.

To analyze microglial motility, subtractions between consecutive Z-stack projections were performed. The number of summed pixels in subtracted images determined the global motility coefficient (arbitrary unit). This coefficient was normalized to the volume of the stack.

### *Ex vivo* experiments

#### Treatment conditions

Three experimental groups were considered. In the anesthesia group, n = 6 mice were injected intraperitoneally with either with a mixture of ketamine (100mg/kg) and xylazine (10mg/kg) or thiopental (60mg/kg) dissolved in 0.9% saline and were euthanized by cervical dislocation 5 minutes after anesthesia, followed by collection of the brain. In the control group, n = 5 mice were injected with an isotonic saline solution (NaCl 0.9%) and were euthanized by cervical dislocation 5 minutes after injection followed by brain collection. Cervical dislocation was authorized by the Ethics Committee of the Institute Pasteur and the French Ministry of National Education and Research in order to avoid any molecular interaction with any type of anesthesia or with carbon dioxide before brain analysis.

#### Tissue preparation

After euthanasia by cervical dislocation, the brains were immediately removed and sectioned along the inter-hemispherical fissure on a sagittal plane. The left hemisphere, dedicated to the morphological analysis, was fixed for 24 hours in a 4% paraformaldehyde solution (QPath®, VWR Chemicals, Fontenay Sous Bois, France) and then stored in a 0.1% paraformaldehyde solution until carrying out floating sections of 80μm along a sagittal plane using a vibratome (VT 1000 S, Leica®, Germany). The most medial section was then used for the morphological analysis.

#### Microglial morphology imaging and analysis

Microglial morphologic criteria were determined with an automated confocal tissue imaging system coupled to morphological modelling in CX3CR1^GFP/+^ transgenic mice. This analysis was performed on sagittal cerebral floating sections of the left hemisphere placed on glass slides with FluoroMount (FluoroMount-G Mounting Medium, FluoProbes).

The image acquisition was carried out according to a previously validated protocol [24] using a confocal spinning disk microscope (Cell Voyager—CV1000, Yokogawa®, Japan) equipped with a UPLSAPO objective 40x/NA 0.9. Automatic analysis was applied using analysis script developed with the image analysis software Acapella™ (version 2.7—Perkin Elmer Technologies, Waltham, USA). The following morphological criteria have been defined for each microglial cell on more than 3,000 microglial cells by group: the area of the cell body and the cytoplasmic area, defined as the area of the cytoplasm included in the primary branches, expressed in μm^2^; a second set of calculated criteria extrapolated from the previous ones yielded the complexity index (CI) and the covered environment area (CEA). We defined the CI by the ratio between the number of segments of each ramification of each cell multiplied by the sum of the nodes on one hand and the number of primary branches on the other hand. Thus, we obtained an average complexity relative to the number of primary branches for each microglial cell.

$$CI=\frac{nb\;of\;segments\times (nb\;of\;nodes\;1+nb\;of\;nodes\;2)}{nb\;of\;roots}$$

The CEA represents the 2D total surface covered by its ramifications and defined as the area of the polygon formed by linking the extremities of its processes, expressed in μm^2^.

### Statistical analysis

All statistical analyses were performed using the Prism V statistical analysis software (GraphPad, La Jolla, Ca). For *in vivo* experiments, microglial complexity and motility index were compared between groups using two-tailed paired t-test. The normal distribution of data was examined using d’Agostino-Pearson test.

For *ex vivo* experiments, we assessed a potential microglial effect of ketamine/xylazine or barbiturates using the Kruskal-Wallis test after the Shapiro-Wilk test showed a non-normal distribution of the data. The alpha-level of 0.05 was adjusted for the number of comparisons to control for family-wise error. When multiple comparisons were needed, analysis using Sidak’s corrections were realized. Significant comparisons between groups are indicated in the figures. Significance of p<0.05 was used for all analyses.

## Results

### Ketamine/xylazine and pentobarbital administration generate distinct and specific patterns of neuronal activity *in vivo*

**Fig 1:**
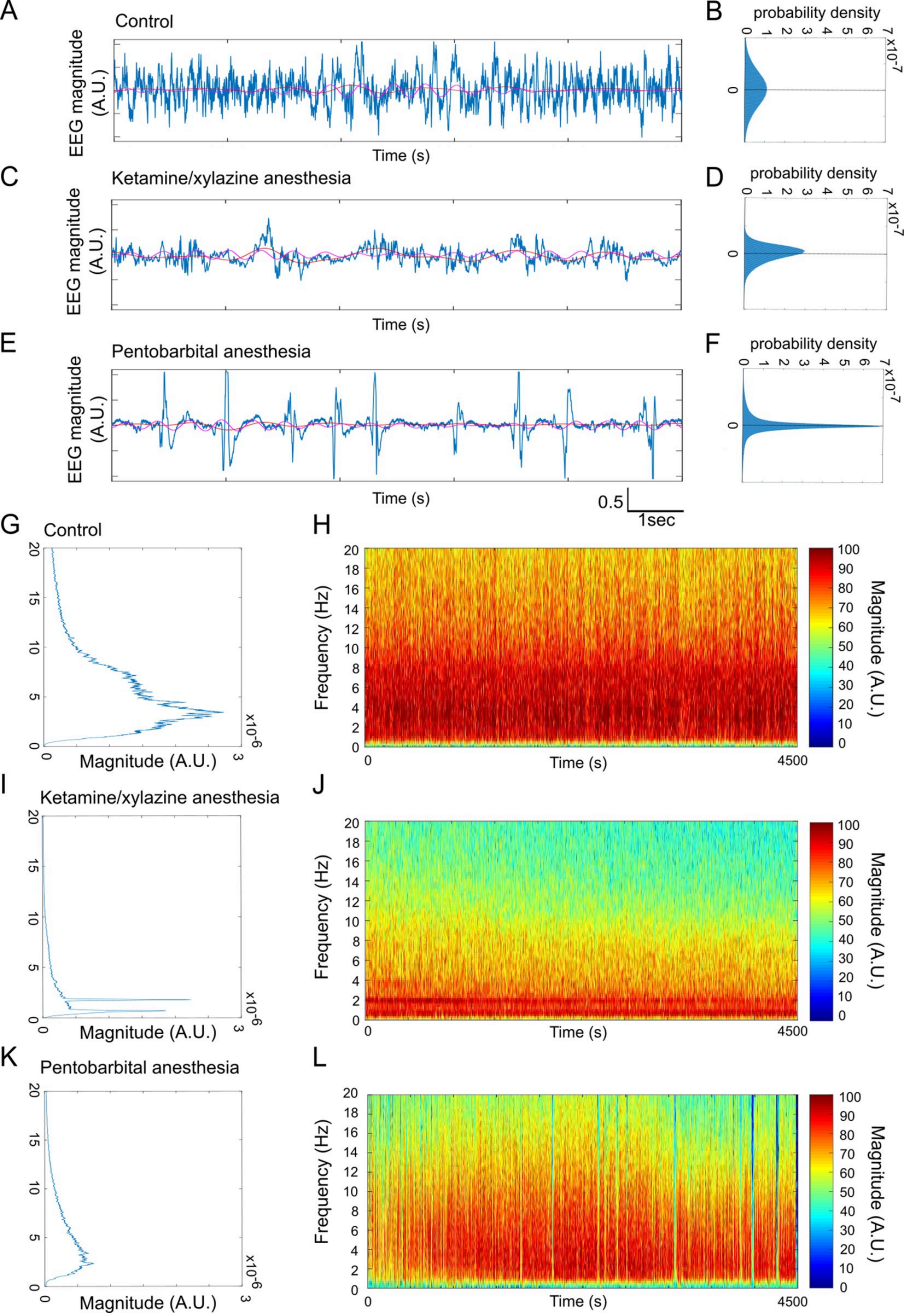
Ketamine/xylazine and pentobarbital anesthesia are associated with different patterns of neuronal activity. (A-F) Examples of EEG traces (A, C, E) and their corresponding amplitude distributions (B, D, F) in different conditions: control (A, B), during ketamine/xylazine (C, D) and pentobarbital (E, F) anesthesia. (G-L) Characteristic power spectrum (G, I, K) and normalized color-coded logarithmic amplitude of time-frequency graphs (H, J, L) in control condition (G, H), and during ketamine-xylazine (I, J) and pentobarbital (K, L) anesthesia.

Anesthetic effects on global neuronal activity were monitored using EEG/EMG recordings. EEG signal patterns were different between vigilance states and varied depending on the anesthetic agent (Fig 1A, 1C and 1E). The unanaesthetized condition is characterized with a large spectral range (Width to Mid-Height: WHM) equal to 10Hz, as well as a high dispersion of the amplitudes’ distribution (Fig 1A, 1B, 1G and 1H). Ketamine/xylazine anesthesia was characterized with slow and large amplitude waves (Fig 1C and 1D) close to pure bi-chromatic signal (0.5Hz and 2Hz, Fig 1I) continuously present during the anesthesia period (Fig 1J). The 0.5 Hz component is stable during anesthesia while the 2Hz component tends to vanish at the end of the recoded period (Fig 1J). Pentobarbital anesthesia caused states of low electric activity with sporadic bursts of high amplitude (Fig 1E), consistent with the high peak at 0 (around baseline) and the long wings observed on the amplitudes’ distribution (Fig 1F). The EEG signal exhibited a more spread out spectrum in comparison to ketamine/xylazine (Fig 1I and 1J). The time-frequency representation of pentobarbital anesthesia (Fig 1K and 1L) showed a gradual increase of the spectral range before continuously decreasing until the end of the recording.

### Both anesthetics reduce microglial motility, but only ketamine/xylazine affects microglial morphology *in vivo*

**Fig 2:**
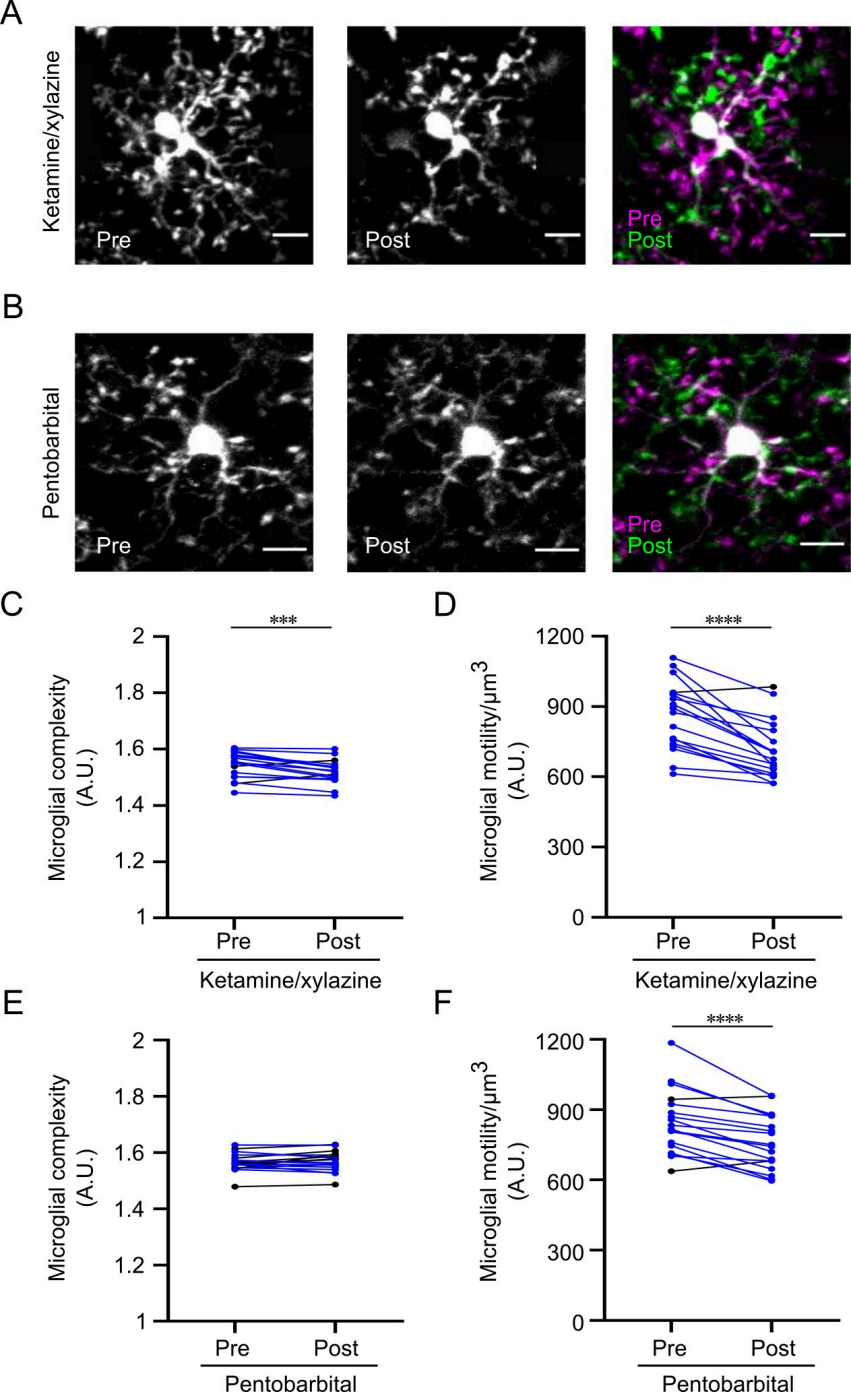
Ketamine/xylazine and pentobarbital anesthesia reduce microglial motility *in vivo*, while microglial complexity is reduced by ketamine/xylazine only. (A, B) Individual and color-coded representative images of microglial cells pre- and post- ketamine/xylazine anesthesia (A) and pentobarbital (B) anesthesia. For quality purposes, brightness and contrast were enhanced similarly for the two sets of images. The scale bars equal 10μm. (C, D) Quantification of microglial complexity (C) and motility (D) pre- and post- ketamine/xylazine anesthesia (n = 18 cells, with 3 microglia analyzed per mouse, two-tailed paired t-test). Microglial cells for which these parameters decrease during anesthesia are represented in blue. (E, F) Quantification of microglial complexity (E) and motility (F) pre- and post- pentobarbital anesthesia (n = 18 cells, with 3 microglia analyzed per mouse, two tailed paired t-test). Microglial cells for which these parameters decrease during anesthesia are represented in blue. Bars represent mean±SEM. *p<0.05.

We examined, first, microglial morphology, particularly the degree of ramification, assessed by an overall complexity index, and, second, the motility of microglial processes *in vivo* in the somatosensory cortex of CX3CR1^eGFP/+^ mice. These cellular parameters where compared in unanaesthetized and anesthetized conditions. We found that microglial complexity was significantly reduced *in vivo* when mice were injected with ketamine/xylazine (1.545±0.011 vs 1.52±0.01, p<0.001; Fig 2A and 2C). However, microglial complexity remained unaltered with thiopental administration (1.565±0.008 vs 1.568±0.008, p>0.05; Fig 2B and 2E). To address the impact of anesthesia on microglial motility, we recorded 3D time-lapse videos of the same microglial cells when the mouse was not anesthetized and subsequently during ketamine/xylazine or pentobarbital anesthesia. Both ketamine/xylazine and pentobarbital administration resulted in a significantly reduced overall process motility when compared to control (860.73±34 vs 714.82±29.2 motility index/μm^3^ for ketamine/xylazine and 846.3±32 vs 761.76±27.54 motility index/μm^3^ for pentobarbital; p<0.0001; Fig 2D and 2F).

### Ketamine/xylazine and thiopental affect different parameters of microglial morphology *ex vivo*, with inter-regional variability

**Fig 3:**
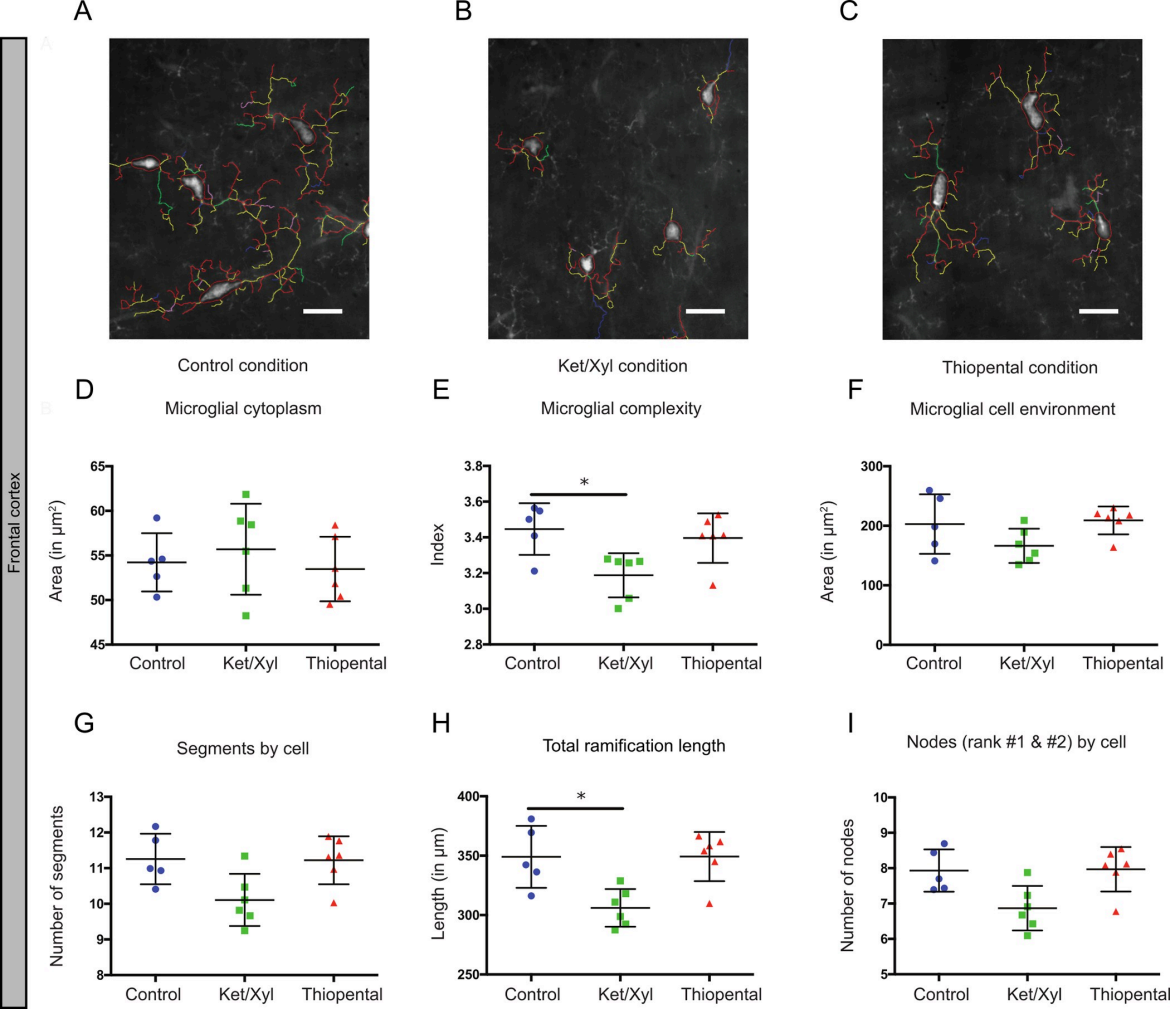
Frontal cortex variability by morphological criteria in the different conditions. (A-C) Microglial modelization characterizing a representative panel of microglial cells in each condition in the frontal cortex. The scale bars equal 10μm. (D-I) Microglial morphology was characterized using the following parameters: microglial cytoplasm (D), the complexity index (E), the cell environment area in μm^2^ (F), the number of segments by cell (G), the total ramification length in μm (H) and the number of nodes (rank #1 & #2) by cell (I). Data shown are mean±SD in the control, ketamine/xylazine and thiopental conditions (n = 5, n = 6 and n = 6, respectively). In CX3CR1^GFP/+^ mice, 305 to 582 microglial cells were analyzed by region and by animal, resulting in studying respectively n = 1975, 2662 and 2495 cells by condition. ANOVA Kruskal-Wallis test was used to compare the different regions. *p<0.05.

**Fig 4:**
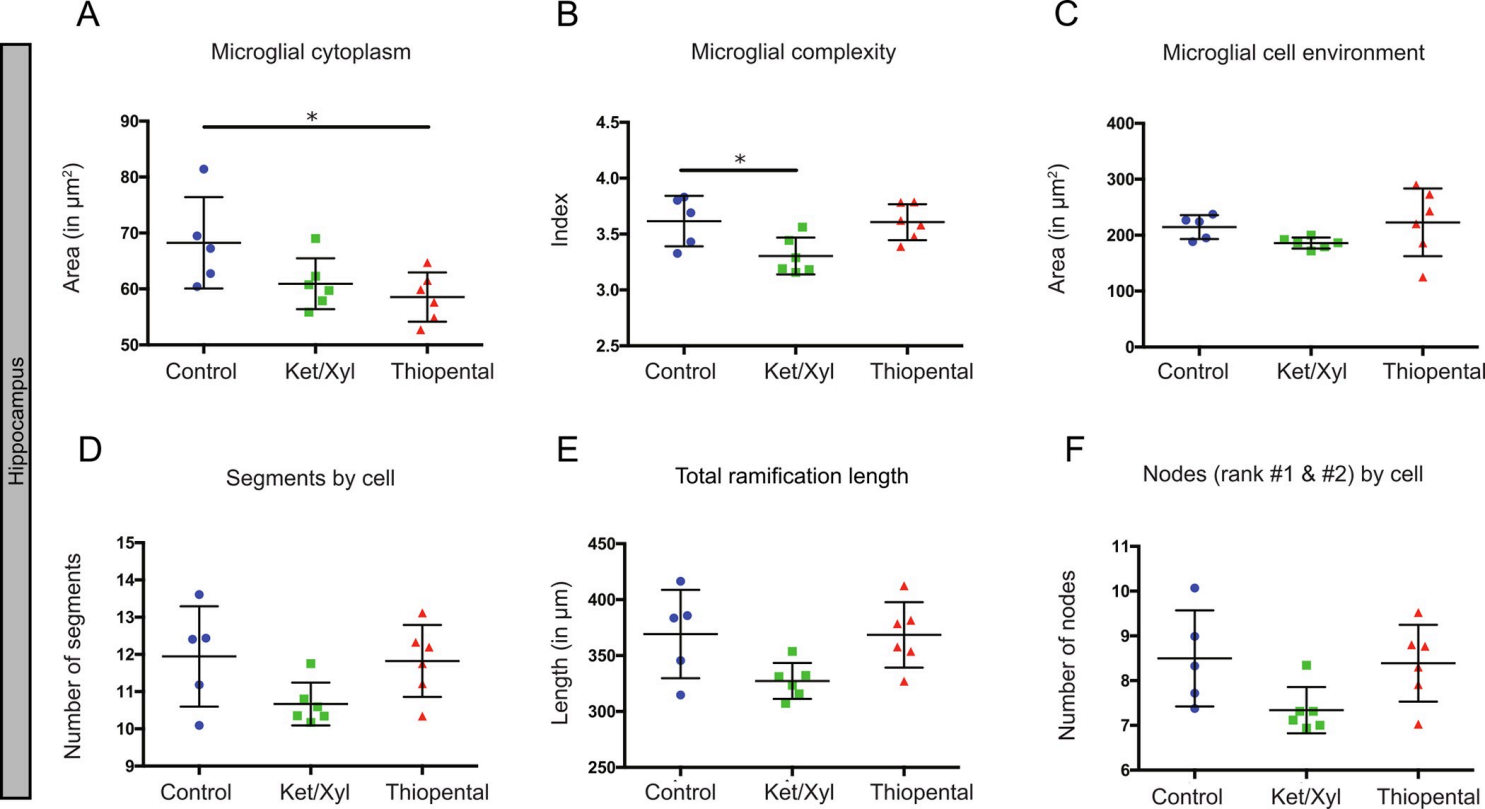
Hippocampus variability by morphological criteria in the different conditions. Microglial morphology was characterized using the following parameters: microglial cytoplasm (A), the complexity index (B), the cell environment area in μm^2^ (C), the number of segments by cell (D), the total ramification length in μm (E) and the number of nodes (rank #1 & #2) by cell (F). Data shown are mean±SD in the control, ketamine/xylazine and thiopental conditions (n  =  5, n = 6 and n  =  6, respectively). In CX3CR1^GFP/+^ mice, 272 to 448 microglial cells were analyzed by region and by animal, resulting in studying respectively n = 1713, 1808 and 2173 cells by condition. ANOVA Kruskal-Wallis test was used to compare the different regions. *p<0.05.

To further describe the morphological changes of microglia and evaluate their heterogeneity in different brain areas, we studied microglial morphology in brain sections from CX3CR1^GFP/+^ mice administered with ketamine/xylazine, thiopental anesthesia or saline prior to euthanasia (Figs 3 and 4).

We quantified these morphological parameters in microglial cells separately in the frontal cortex (Fig 3) and the hippocampus (Fig 4). In both regions, the complexity of microglial cells (3.2±0.1 vs 3.4±0.1 for the frontal cortex and 3.3±0.2 vs 3.6±0.2 for the hippocampus; p<0.05, Figs 3E and 4B) were significantly reduced with ketamine/xylazine anesthesia and not with thiopental. However, we observed a significant decrease of the total length of the ramifications in the frontal cortex only (306±16 vs 349±26 μm; p<0.05; Fig 4E). Treatment with thiopental affected exclusively the cytoplasm area in the hippocampus but not in the frontal cortex (58.5±4.4 vs 68.3±8.2 μm^2^; p<0.05; Figs 3D and 4A), which was not the case upon ketamine/xylazine administration. Other morphological features such as the cell environment covered by the ramifications and the number of segments remained unchanged in both conditions and both regions.

## Discussion

Our current findings demonstrate that administration of two commonly used *iv* anesthetics in clinic and in research protocols, ketamine/xylazine and barbiturates, resulted in microglial surveillance reduction *in vivo* and morphological alterations that depended on the type of anesthetics administered and the brain region examined (S1 Table). Ketamine/xylazine administration resulted in extensive and widespread reduction of microglial process complexity, whereas barbiturates affected the cytoplasm area in a limited manner.

Ketamine/xylazine and barbiturates are commonly used as general anesthetics for surgery, imaging, or euthanasia preceding immunohistochemistry studies. For anesthesia, ketamine, a NMDAR antagonist is often used with an α_2_ adrenergic agonist, in our case xylazine, which provides sedation and analgesia [25,26]. Importantly, ketamine activates less GABA_A_R in comparison with other anesthetics, which allowed us to distinguish the effects of ketamine/xylazine and barbiturates that are GABA_A_ agonists [27]. For the *ex vivo* and *in vivo* experiments, we used two types of barbiturates, an oxybarbiturate, i.e. pentobarbital, and thiobarbiturate, i.e. thiopental, characterized by different duration of action but presenting similar chemical structure and functions. Both of them act mainly by activating the gamma-aminobutyric acid A (GABA_A_) receptors, keeping the chlorine channel open, resulting in hyperpolarization of the post-synaptic membrane [28]. In the same manner, previous studies have shown that thiopental administration leads to changes similar to pentobarbital-induced EEG alterations, including burst suppression activity [29]. Thiopental is an ultra-short acting anesthetic that was used for *ex vivo* experiments whereas pentobarbital was preferred for the *in vivo* study because its action lasts 4 to 8 times longer than thiopental [30].

Interestingly, different anesthetics generate distinct and specific patterns of neuronal activity. Our EEG recordings indicate that ketamine/xylazine anesthesia is characterized by slow, large amplitude waves with high delta power. More precise LFP and intracellular recordings showed that ketamine/xylazine administration results in long duration of silent states and increased gamma activity power [2]. On the other hand, pentobarbital anesthesia was associated with a different EEG pattern consisting in states of isoelectric activity with bursts of high-amplitude activity. It has already been shown that propofol, isoflurane and the barbiturate thiopental led to high-amplitude burst suppression activity separated by brief episodes of isoelectric activity [31]. Interestingly, in our study, the anesthetic inducing the greatest change in activity in comparison to wake also induced major microglial morphology and dynamics change. This possibility is supported by a recent study showing that inducing neural spiking activity at 40Hz leads to morphological changes of microglia [32]. These findings need further investigation, to determine whether and how different EEG patterns, in terms of frequency and amplitude, may impact microglial morphology and motility.

Microglial cells continuously survey their surroundings by extending and retracting their motile processes [7,33]. They make direct contacts with synapses that seem to be dependent, at least partly, on neuronal activity [8,9,11,34]. We found that both anesthetics induced a reduction of microglial motility in the somatosensory cortex. Our results are thus in line with previous studies showing that blocking NMDAR-mediated glutamatergic transmission induced a significant decrease in microglial motility in retinal explants (−12.5% decrease) while GABA application decreased microglial motility as well (−7.9% decrease) [35]. However, the reduction that we observed with ketamine/xylazine does not match a recent study that reports no effect of ketamine/xylazine anesthesia on microglial motility [13]. Even though both studies were performed *in vivo*, several discrepancies should be highlighted: 1) we used a thin-skull cranial preparation, which is more immunologically inert compared to cranial window preparation used by this study; 2) Sun *et al*. imaged different microglia from the two hemispheres with 2-h rest between conditions, while we imaged the same microglia in presence and absence of anesthetics within a shorter time lapse, which we believe adds to the precision of the measurements.

To date, there is no consensus with regards to the effect of anesthetics on microglial motility and it may well be dependent on the anesthetic agent. For instance, a recent study showed that isoflurane administration resulted in increased process velocity [5], while fentanyl anesthesia decreased overall motility [14]. Importantly, the last study reported that the effects of fentanyl cocktail on motility were quite complex since it increased the motility of terminal processes but decreased overall motility due to the loss of motile filopodia. Therefore, future research needs to take into consideration the different aspects of microglial motility.

It is unknown whether the anesthetics we used have a direct action on microglial cells. Indeed, various evidence report the presence of glutamate and GABA receptors on microglial surface [36,37]. However microglia lack electrical responses to local application of GABA and glutamate in retinal slices [35], as well as to puffs of glutamate or NMDA in hippocampal slices [34] casting doubt on a direct action on microglia. The observed effect could also be due to the action of anesthetics on sites other than their major target receptors. For example, pentobarbital also targets alpha-amino-3-hydroxy-5-methyl-4-isoxazolepropionic acid receptors (AMPAR) [38,39] and voltage-dependent Na^+^ channels, while ketamine affects also the cholinergic muscarinic receptors (antagonistic effect) [40,41] and α(alpha) and β(beta) adrenergic receptors (agonistic effect) [42]. Ketamine and pentobarbital could also target ion channels expressed by microglial cells and alter membrane properties [43,44]. A recent study found a direct impact of gaseous anesthetics on microglial motility by action on tandem pore domain halothane inhibited K(+) channel (THIK)-1, a two-pore domain K^+^ channel present on microglial cells [12]. Likewise, possible changes in extracellular ion concentration due to altered neurotransmission by anesthesia might affect microglial resting potential and consequently microglial motility. Anesthetics could also modulate microglia through indirect action. For example, extracellular nucleotides, in particular adenosine triphosphate (ATP), elicit membrane currents in microglial cells via ionotropic (P2X) and metabotropic (P2Y) purinergic receptors and affect microglial motility [34,35,45–48]. Importantly, ATP is co-released with the main transmitters from neurons [49] and from astrocytes [50] at synapses in response to neuronal activity. ATP released could lead to a chemotactism of microglial processes toward highly active spines [11] possibly by a NMDAR-dependent ATP release [34]. Thus, a reduction of ATP release caused by reduction of neuronal activity may decrease microglial motility. Finally, both *IV* and inhaled anesthetics have been shown to disrupt astrocyte calcium signaling in the cortex [4] and consequently the calcium-dependent release of ATP that could regulate microglial motility.

Morphological changes of microglial cells are often associated with microglial activation. Indeed, immunohistochemistry studies rely on quantification of microglial morphology to characterize inflammation. We found both *in vivo and ex vivo* that ketamine/xylazine and barbiturates affected microglial morphology in different ways. Ketamine/xylazine anesthesia caused a significant reduction of microglial complexity overall. Previous findings on the effect of ketamine/xylazine on microglial morphology appear quite divergent. Recently, Liu *et al*., reported that ketamine/xylazine administration resulted in increased process area and microglial surveillance territory in the somatosensory cortex [5], while Sun *et al*., found no effect on microglial process length and ramification in cortical microglia [13]. In addition to a salient difference in the imaging paradigms (thin-skull preparation vs open-skull preparation), the discrepancies between these studies may arise from two important points. First, each study uses different approaches for quantifying various aspects of microglial morphology. For instance, even though we report seemingly opposite effects on microglial morphology in the same cortical region with Liu et al., our quantification of microglial complexity is not comparable to their measurement of microglial process area and surveillance territory and therefore both findings are not necessarily contradictory. Another example is that for the quantification of microglial process length, Sun *et al*., takes into consideration only the primary and secondary branches, while our method also includes measurements of higher-order branches. Secondly, we must be aware of inter-regional differences, as highlighted in our study. In the study of Sun et al., microglial cells are investigated in a large field, possibly comprising measurements of microglia from the somatosensory, motor, and visual cortex, which does not take into consideration potential regional differences. In favor of our results, it has previously been found that blocking NMDAR by D-AP5 resulted in significant decreases in all morphological parameters studied, such as the total dendritic length, the total branch point and the dendritic tree area in retinal explants [35]. Because β-adrenergic receptor agonist, isoproterenol, has been shown to induced a considerable decrease in the ramifications of resting microglia in acute slices [51], another effect of ketamine could be through its agonist action on β-adrenergic agonist receptors.

We found no effect of pentobarbital on microglial complexity in the somatosensory cortex *in vivo*. More precise *ex vivo* quantifications found that barbiturates affected only the cytoplasm area of microglial cells in the hippocampus, which is defined as the cell body area associated with the cytoplasmic area of the primary ramifications. In that regard, the study of Fontainhas *et al*. shows that GABA application in retinal explants affected microglial complexity [35]. The discrepancy between these results could be explained by the heterogeneity of microglial cells between retina and the brain. Another explanation could be the use of barbiturates that target specifically GABA_A_ while the administration of GABA might also target GABA_C_ and GABA_B_ receptors.

We assessed changes in microglial morphology induced by anesthetics in the hippocampus and the frontal cortex, two areas where microglial cells contribute to neuropsychiatric disorders such as dementia or depression [52,53]. Ketamine/xylazine caused a significant reduction in microglial complexity in both regions, accompanied with a reduction in microglial ramification length in the frontal cortex only. This reduction was not accompanied by a significant reduction in the number of segments and nodes rank 1 and 2 suggesting that ketamine/xylazine induced distal microglial ramification modifications. The effects of thiopental also differed depending on the brain region. Thiopental caused a reduction in the cytoplasm area of the hippocampus, but not the frontal cortex. The discrepancy between the effects of anesthetics on microglial cells in these different regions may come from several reasons. First, the composition and function of glutamatergic and GABAergic networks may vary between the hippocampus and cortex. Second, the neuronal NMDA and GABA_A_ receptor subtype composition may differ between areas. Third, microglial phenotypic heterogeneity: microglial expression profiles, receptor and channel distribution, and the resting potential of microglial cells may vary between different regions [54–57], thus contributing to different responses of microglial cells.

## Conclusions

Overall, our study shows that both anesthetics reduced microglial motility. Ketamine/xylazine had a greater effect on microglial morphology, whereas the effect of barbiturates was limited to the cytoplasm area. Even though both anesthetics alter microglial motility, barbiturates might be appropriate anesthetic agents for the study of microglial morphology, at least for histochemical studies.

Our findings have major implications for research studies. Many *ex vivo* studies are based on the characterization of microglial morphology to evaluate the activation status and inflammatory profile of microglial cells and may thus be biased by the type of anesthetic used. Furthermore, the disclosed alterations of microglial motility and morphology may have unintended consequences on microglial responses *in vivo* and bias experimental results. Future studies need to assess the potential alterations of additional parameters associated with microglial morphology and motility under anesthesia. These include microglial interaction with spines and neuronal networks, the phagocytic capacity of microglial cells, and their cytokine secretion. Furthermore, it is important to determine whether anesthetics maintain their effects on microglial cells’ morphology and motility *in vivo* past anesthesia and the potential long-term effects. If this is the case, multiple imaging sessions with repeated animal exposure to anesthesia may have serious consequences on the experimental results. Finally, since the developing brain and the aging brain are more vulnerable to anesthesia-induced neurotoxicity, we need to study the short-term and long-term effects of anesthetic agents on microglial cells in these populations.

## Supporting information

S1 Table Summary of the findings from the *in vivo* and *ex vivo* studies of the impact of ketamine/xylazine and barbiturates on microglial motility and several parameters of microglial morphology. “Ns” and “↙” correspond to non-significant change and significant decrease, respectively. The “↙” corresponds to a significant reduction p<0.05.
(PDF)

S2 Table Minimal data set for Figs 2–4. (PDF)
