# Supplementary material for: Ketamine/xylazine and barbiturates modulate microglial morphology and motility differently in a mouse model
Source: PLoS One. 2020 Aug 6;15(8):e0236594. doi: 10.1371/journal.pone.0236594 (PMC7410236; doi:10.1371/journal.pone.0236594)
Supplement: S2 Table — (PDF) [file pone.0236594.s002.pdf]

Fig 2C	Microglial complexity	
	Pre-ket/xyl	Post-ket/xyl
Values	1.589813563	1.535612357
	1.598335304	1.584283553
	1.539948937	1.560683102
	1.516786632	1.490205717
	1.57431312	1.538736543
	1.556073862	1.503621982
	1.604381649	1.601622396
	1.502075107	1.495271854
	1.445230362	1.434893783
	1.594030138	1.533133872
	1.579194713	1.546303997
	1.568386249	1.520518006
	1.477742716	1.511342142
	1.502107789	1.492477183
	1.481255801	1.447081837
	1.57948573	1.53434315
	1.550472439	1.498137048
	1.553221984	1.524583994
**Mean**	**1.545158672**	**1.519602918**
**SEM**	**0.011123085**	**0.009869768**
**Statistical method**	Paired t-test, two tailed	
**p-value**	***p<0.001	
**# cells**	18	18

Fig 2D	Microglial motility	
	Pre-ket/xyl	Post-ket/xyl
Values	741.1652524	633.6537545
	874.3865943	798.2409871
	960.3261924	984.7037826
	764.9635285	572.0439496
	893.5543853	706.0208525
	733.3180186	601.7652104
	814.271851	675.2949477
	933.2632106	852.8060132
	612.4491291	571.1101922
	1046.914288	646.6579845
	958.4653837	823.4860105
	758.0204792	655.6078447
	638.6186698	609.5658157
	719.7512485	614.9074623
	909.1818296	709.9264306
	1108.854035	954.0874585
	1074.250903	750.2197091
	951.3645108	706.5891
**Mean**	**860.7288617**	**714.8159725**
**SEM**	**34.39918115**	**29.19523509**
**Statistical method**	Paired t-test, two tailed	
**p-value**	****p<0.0001	
**# cells**	18	18

Fig 2E	Microglial complexity	
	Pre-pento	Post-pento
Values	1.479133065	1.48639672
	1.546229554	1.539486458
	1.54573941	1.557500897
	1.603696273	1.580118169
	1.558112188	1.526161703
	1.627189589	1.626023184
	1.56064275	1.555195613
	1.565231482	1.586279565
	1.542170597	1.594389887
	1.572623602	1.559559698
	1.561611859	1.575038664
	1.571754227	1.563928153
	1.556566274	1.578045862
	1.580052777	1.606503394
	1.556303117	1.54765312
	1.591221344	1.591554174
	1.539678727	1.528475888
	1.613333483	1.628736904
**Mean**	**1.565071684**	**1.568391559**
**SEM**	**0.007717446**	**0.00848031**
**Statistical method**	Paired t-test, two tailed	
**p-value**	p>0.05	
**# cells**	18	18

Fig 2F	Microglial motility	
	Pre-pento	Post-pento
Values	701.1620645	681.4787444
	857.7296314	720.3899371
	816.4977289	688.5277835
	1010.027849	880.8181989
	746.1730688	601.5994777
	1021.288604	873.2763583
	760.4657055	647.9059597
	869.3778987	809.1606861
	637.9018478	681.7006905
	1185.767106	960.3784895
	887.3529871	827.5206656
	708.9661404	617.1317003
	833.8512766	799.0240303
	923.6451938	873.2442586
	808.6262107	751.7762988
	944.8154434	957.3760493
	714.1810186	596.6062315
	805.5660382	743.6784492
**Mean**	**846.2997674**	**761.7552227**
**SEM**	**32.00374675**	**27.54474249**
**Statistical method**	Paired t-test, two tailed	
**p-value**	****p<0.0001	
**# cells**	18	18

Fig 3D	Microglial cytoplasm		
	Control	Ket/xyl	Thiopental
Values	59.2	48.24	50.4
	52.64	61.84	51.84
	54.6	58.86	49.52
	50.32	55.48	53.56
	54.36	51.32	57.12
		58.44	58.4
**Mean**	**54.224**	**55.69666667**	**53.4733333**
**SD**	**3.266141454**	**5.100531998**	**3.6144082**
**Statistical method**	Kruskall Wallis test and Dunn's correction		
**p-value**		p>0.05	p>0.05
**# cells**	1975	2662	2495

Fig 3E	Microglial complexity		
	Control	Ket/xyl	Thiopental
Values	3.211	3.001	3.411
	3.501	3.265	3.409
	3.548	3.264	3.489
	3.564	3.257	3.132
	3.409	3.059	3.527
		3.279	3.408
**Mean**	**3.4466**	**3.1875**	**3.396**
**SD**	**0.14486649**	**0.123576292**	**0.13859582**
**Statistical method**	Kruskall Wallis test and Dunn's correction		
**p-value**		p<0.05	p>0.05
**# cells**	1975	2662	2495

Fig 3F	Microglial cell environment		
	Control	Ket/xyl	Thiopental
Values	198.4	134.8	213.4
	259.3	169	230.3
	245.9	189.4	208.4
	141	154.1	163.8
	169.6	141.9	220.3
		209	217.9
**Mean**	**202.84**	**166.3666667**	**209.016667**
**SD**	**49.97672458**	**28.6377839**	**23.338159**
**Statistical method**	Kruskall Wallis test and Dunn's correction		
**p-value**		p>0.05	p>0.05
**# cells**	1975	2662	2495

Fig 3G	Segments by cell		
	Control	Ket/xyl	Thiopental
Values	10.93	9.246	10.97
	12.17	10.11	11.36
	11.78	10.47	11.31
	10.41	9.817	10.03
	10.99	9.666	11.89
		11.34	11.77
**Mean**	**11.256**	**10.10816667**	**11.2216667**
**SD**	**0.70758745**	**0.731021318**	**0.67196478**
**Statistical method**	Kruskall Wallis test and Dunn's correction		
**p-value**		p>0.05	p>0.05
**# cells**	1975	2662	2495

Fig 3H	Microglial ramification length		
	Control	Ket/xyl	Thiopental
Values	336.3	287.6	345
	380.9	310.9	358.3
	369.4	328.8	354.1
	316.2	298.8	309.8
	342.3	292.4	361.8
		318	366.6
**Mean**	**349.02**	**306.0833333**	**349.266667**
**SD**	**26.05661145**	**15.89445396**	**20.6800064**
**Statistical method**	Kruskall Wallis test and Dunn's correction		
**p-value**		p<0.05	p>0.05
**# cells**	1975	2662	2495

Fig 3I	Microglial nodes		
	Control	Ket/xyl	Thiopental
Values	7.435	6.093	7.885
	8.694	6.909	8.117
	8.439	7.23	8.077
	7.395	6.679	6.779
	7.698	6.425	8.55
		7.877	8.399
**Mean**	**7.9322**	**6.868833333**	**7.96783333**
**SD**	**0.597458534**	**0.62982487**	**0.62915449**
**Statistical method**	Kruskall Wallis test and Dunn's correction		
**p-value**		p>0.05	p>0.05
**# cells**	1975	2662	2495

Fig 4A	Microglial cytoplasm		
	Control	Ket/xyl	Thiopental
Values	60.4	55.86	54.86
	67.24	60.76	61.5
	81.4	59.72	59.88
	62.76	62.3	52.72
	69.48	57.88	57.62
		69	64.72
**Mean**	**68.256**	**60.92**	**58.55**
**SD**	**8.173400761**	**4.549760433**	**4.40477468**
**Statistical method**	Kruskall Wallis test and Dunn's correction		
**p-value**		p>0.05	p<0.05
**# cells**	1713	1808	2173

Fig 4B	Microglial complexity		
	Control	Ket/xyl	Thiopental
Values	3.328	3.182	3.389
	3.691	3.444	3.623
	3.831	3.191	3.784
	3.43	3.157	3.581
	3.802	3.29	3.477
		3.563	3.787
**Mean**	**3.6164**	**3.3045**	**3.60683333**
**SD**	**0.225823604**	**0.165007576**	**0.16067659**
**Statistical method**	Kruskall Wallis test and Dunn's correction		
**p-value**		p<0.05	p>0.05
**# cells**	1713	1808	2173

Fig 4C	Microglial cell environment		
	Control	Ket/xyl	Thiopental
Values	188.6	171.7	125.1
	237.4	186.5	220.6
	226.8	192	273.2
	195	179.1	186.1
	224	200	243.2
		186.5	289.7
**Mean**	**214.36**	**185.9666667**	**222.983333**
**SD**	**21.31262537**	**9.844727861**	**60.5454347**
**Statistical method**	Kruskall Wallis test and Dunn's correction		
**p-value**		p>0.05	p>0.05
**# cells**	1713	1808	2173

Fig 4D	Segments by cell		
	Control	Ket/xyl	Thiopental
Values	10.09	10.17	10.34
	12.44	10.35	12.2
	13.61	10.8	12.33
	11.18	10.34	11.21
	12.41	10.59	11.75
		11.75	13.12
**Mean**	**11.946**	**10.66666667**	**11.825**
**SD**	**1.347193379**	**0.574618714**	**0.96558273**
**Statistical method**	Kruskall Wallis test and Dunn's correction		
**p-value**		p>0.05	p>0.05
**# cells**	1713	1808	2173

Fig 4E	Microglial ramification length		
	Control	Ket/xyl	Thiopental
Values	314.7	307.2	327
	385.8	315.7	378.5
	416.4	331.3	381.6
	345.6	323.4	353.8
	383.6	332.3	357.7
		353.8	412.4
**Mean**	**369.22**	**327.2833333**	**368.5**
**SD**	**39.4904039**	**16.10607546**	**29.1677905**
**Statistical method**	Kruskall Wallis test and Dunn's correction		
**p-value**		p>0.05	p>0.05
**# cells**	1713	1808	2173

Fig 4F	Microglial nodes		
	Control	Ket/xyl	Thiopental
Values	7.378	6.94	7.028
	8.329001	7.117	8.767
	10.07	7.314	8.804
	7.72	7.004	7.909
	8.989	7.314	8.302
		8.344	9.523
**Mean**	**8.4972002**	**7.338833333**	**8.38883333**
**SD**	**1.072439563**	**0.516136578**	**0.85922696**
**Statistical method**	Kruskall Wallis test and Dunn's correction		
**p-value**		p>0.05	p>0.05
**# cells**	1713	1808	2173
